# Supplementary material for: The Effect of Polymer Blends on the In Vitro Release/Degradation and Pharmacokinetics of Moxidectin-Loaded PLGA Microspheres
Source: Int J Mol Sci. 2023 Sep 29;24(19):14729. doi: 10.3390/ijms241914729 (PMC10573114; doi:10.3390/ijms241914729)
Supplement: Supplementary file 1 [file ijms-24-14729-s001.zip › ijms-2603875-supplementary.pdf]

International Journal of
Molecular Sciences

The Effect of Polymer Blends on the In Vitro Release/Degradation and Pharmacokinetics of Moxidectin-loaded PLGA Microspheres

Hongjuan Zhang [1], Zhen Yang [1], Di Wu [1], Baocheng Hao [1], Yu Liu [1], Xuehong Wang [1], Wanxia Pu [1], Yunpeng Yi [1,2], Ruofeng Shang [1,*] and Shengyi Wang [1,*]

1 Key Laboratory of New Animal Drug Project, Gansu Province/Key Laboratory of Veterinary Pharmaceutical Development, Ministry of Agriculture and Rural Affairs/ Lanzhou Institute of Husbandry and Pharmaceutical Sciences of CAAS, Lanzhou 730050, China

2 Shandong Provincial Animal and Poultry Green Health Products Creation Engineering Laboratory, Institute of Poultry Science, Shandong Academy of Agricultural Science, Jinan 250023, China

* Correspondence: shangrf1974@163.com (R.S.); wangshengyi@caas.cn (S.W.); Tel.: +86-0931-2115286; Fax: +86-0931-2115191

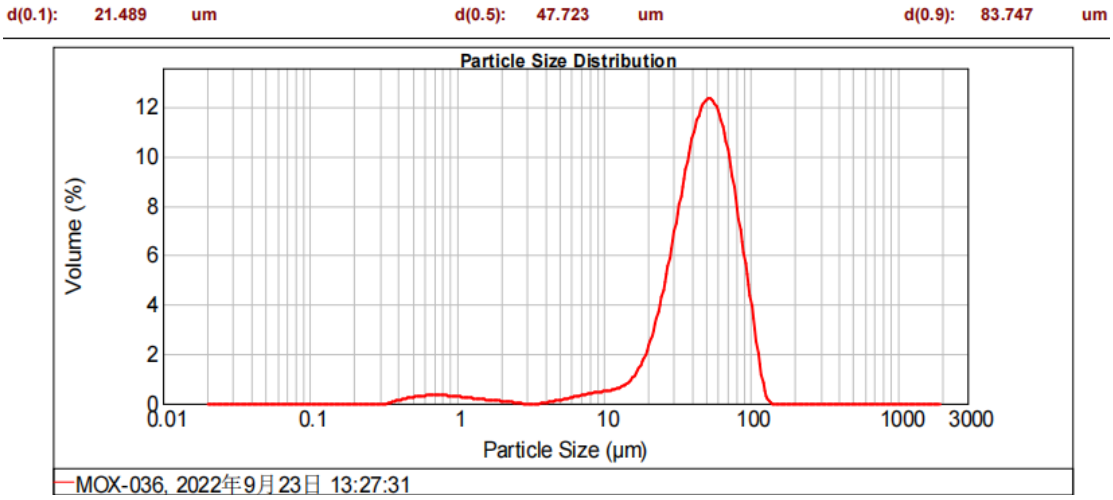

Figure S1. Particle size distribution of F1.

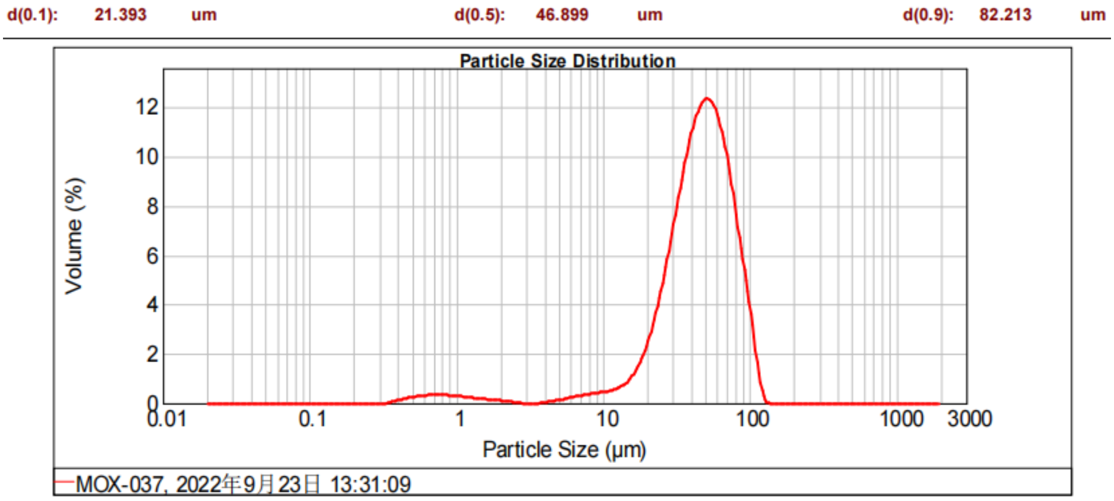

Figure S1. Particle size distribution of F2.

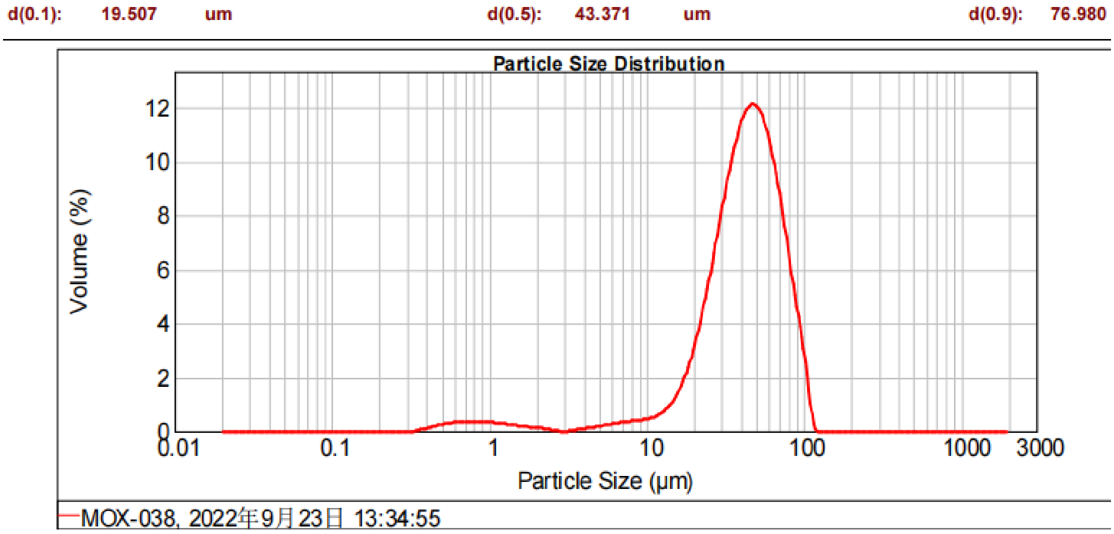

Figure S1. Particle size distribution of F3.

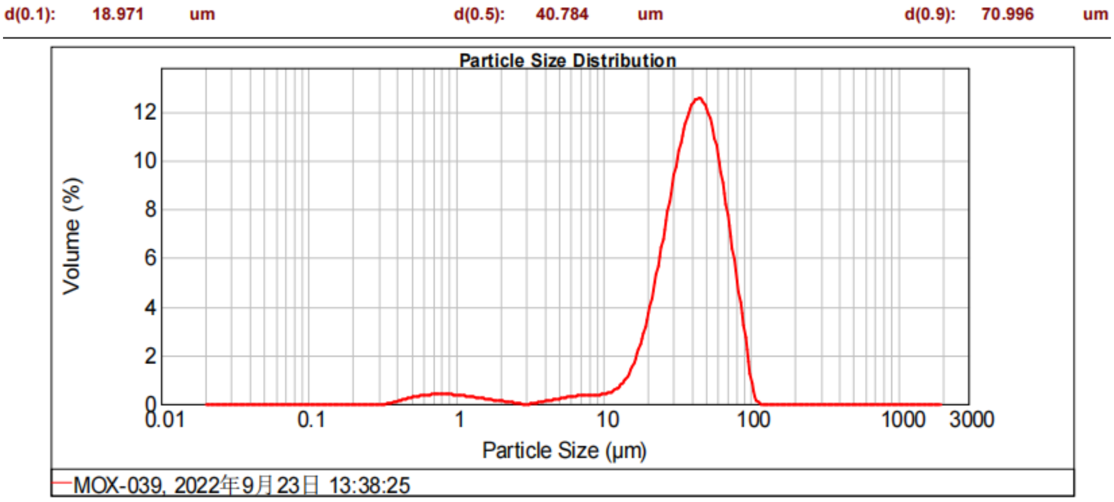

Figure S1. Particle size distribution of F4.

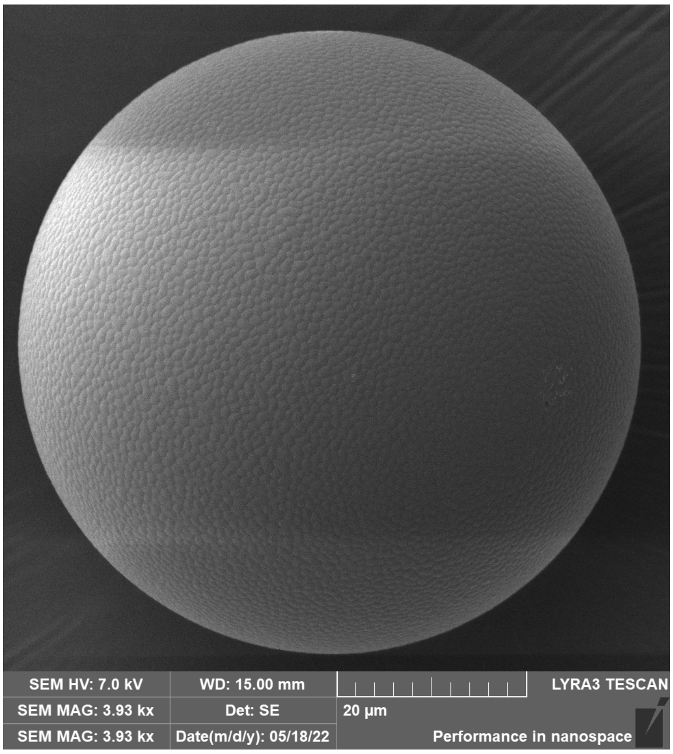

Figure S2. SEM image of MOX-MS (F1).

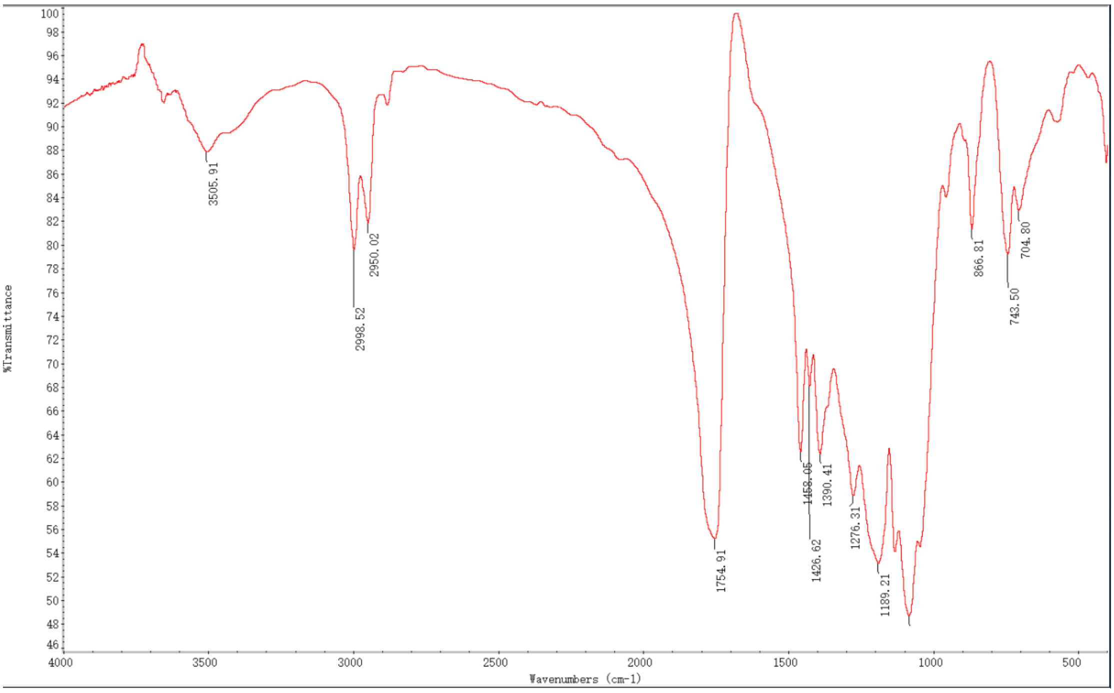

Figure S3. Fourier Transform Infrared (FTIR) spectra of PLGA.

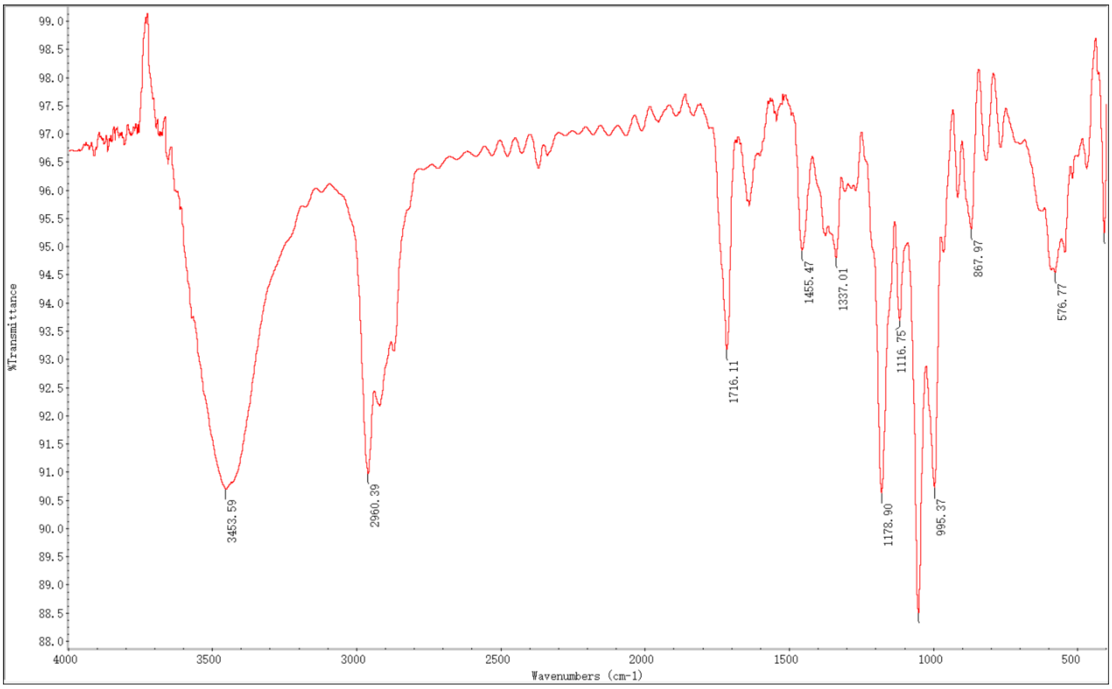

Figure S3. Fourier Transform Infrared (FTIR) spectra of moxidectin.

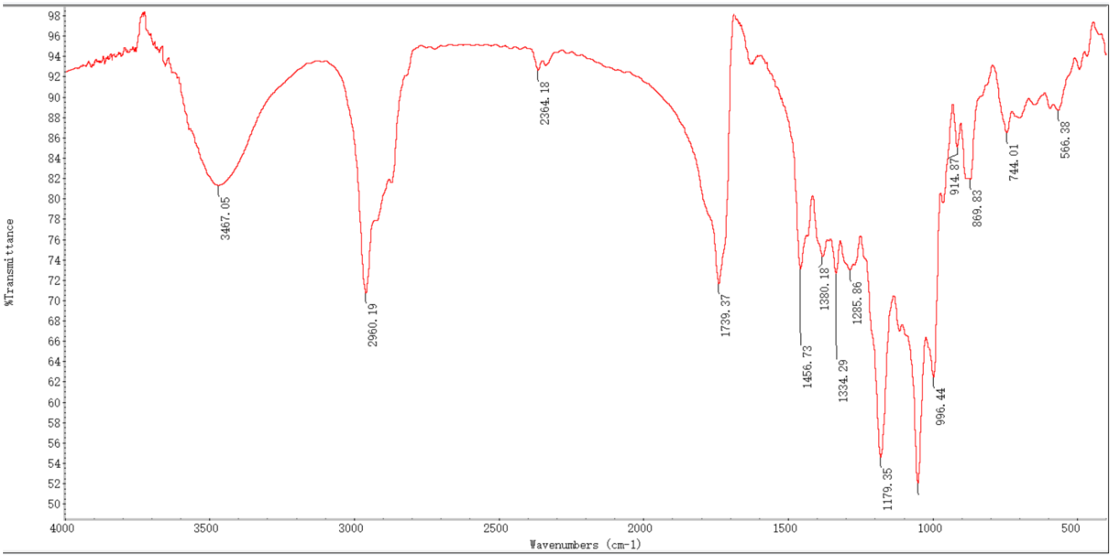

Figure S3. Fourier Transform Infrared (FTIR) spectra of moxidectin loaded microspheres.

Table S1. *In vitro* release values of MOX-MS at 37 °C (n = 3).

Time (d)	F1 ± SD (%)	F2 ± SD (%)	F3 ± SD (%)	F4 ± SD (%)
0.021	5.297 ± 0.289	5.273 ± 0.514	4.857 ± 0.0893	4.267 ± 0.560
0.083	5.369 ± 0.300	5.299 ± 0.663	4.961 ± 0.0799	4.233 ± 0.572
0.167	5.459 ± 0.332	5.429 ± 0.401	5.089 ± 0.0876	4.230 ± 0.474
0.333	5.559 ± 0.329	5.595 ± 0.763	5.163 ± 0.101	4.636 ± 0.267
0.500	5.775 ± 0.131	5.788 ± 0.575	5.393 ±0.288	4.485 ± 0.544
1	5.915 ± 0.964	5.916 ± 0.578	5.554 ± 0.366	4.637 ± 0.469
3	5.862 ± 0.534	5.847 ± 0.300	5.805 ± 0.517	4.646 ± 0.543
7	5.544 ± 0.300	5.601 ± 0.549	5.762 ± 0.333	4.503 ± 0.465
14	5.761 ± 0.299	5.663 ± 0.695	5.832 ± 0.280	4.654 ± 0.414
28	5.760 ± 0.362	5.605 ± 0.804	5.712 ± 0.370	5.073 ± 0.331

49	4.909 ± 0.208	4.821 ± 0.554	5.114 ± 0.340	4.846 ± 0.407
63	4.659 ± 0.281	4.571 ± 0.618	5.358 ± 0.304	12.316 ± 1.112
70	4.591 ± 0.210	4.679 ± 0.462	5.572 ± 0.380	26.324 ± 2.967
77	4.820 ± 0.231	4.695 ± 0.433	9.868 ± 0.847	41.191 ± 1.285
108	8.253 ± 0.309	15.169 ± 0.047	54.362 ± 2.641	91.833 ± 5.890
144	10.138 ± 0.316	65.135 ± 2.878	91.769 ± 4.396	91.327 ± 5.177
164	21.737 ± 0.138	79.723 ± 4.703	90.499 ± 4.801	91.591 ± 3.886
180	59.840 ± 2.185	92.015 ± 4.202	90.205 ± 6.426	90.076 ± 3.592
194	81.789 ± 4.611	91.431 ± 2.331	89.175 ± 6.204	88.365 ± 3.373
220	91.987 ± 5.144	91.761 ± 3.318	88.166 ± 4.476	87.108 ± 3.222
238	91.989 ± 4.960	90.981 ± 1.649	88.000 ± 3.710	87.021 ± 2.190

Table S2. The accelerated *in vitro* release values of MOX-MS at 50 °C (n = 3).

Time (d)	F1 ± SD (%)	F2 ± SD (%)	F3 ± SD (%)	F4 ± SD (%)
0.042	5.427 ± 0.181	4.935 ± 0.095	4.876 ± 0.430	4.232 ± 0.381
0.167	5.822 ± 0.261	5.499 ± 0.198	5.652 ± 0.213	5.054 ± 0.426
0.333	6.517 ± 0.477	6.097 ± 0.209	6.547 ± 0.347	6.019 ± 0.367
1	7.356 ± 0.561	6.792 ± 0.384	8.112 ± 0.221	8.087 ± 0.859
3	9.764 ± 0.536	10.153 ± 0.349	12.215 ± 0.370	13.303 ± 1.240
5	11.593 ± 0.630	12.066 ± 0.672	15.186 ± 0.754	16.369 ± 1.201
7	13.468 ± 0.776	14.260 ± 0.572	17.373 ± 0.935	18.509 ± 1.143
10	15.851 ± 0.851	16.648 ± 0.251	21.103 ± 0.786	23.475 ± 1.505
14	20.297 ± 0.730	21.994 ± 0.726	37.457 ± 1.040	69.418 ± 6.652
18	28.268 ± 0.935	41.492 ± 2.680	88.791 ± 1.424	91.176 ±5.241
22	70.709 ± 1.903	88.725 ± 8.838	95.848 ± 2.195	94.583 ± 5.537
24	92.791 ± 6.176	98.594 ± 0.702	95.827 ± 3.431	94.822 ± 5.022
28	100.900 ± 3.237	96.310 ± 0.354	94.811 ± 4.035	93.748 ± 5.480
35	102.133 ± 3.338	95.909 ± 0.830	94.544 ± 4.268	92.927 ± 5.062

Table S3. The accelerated *in vitro* release values of MOX-MS at 60 °C (n = 3).

Time (d)	F1 ± SD (%)	F2 ± SD (%)	F3 ± SD (%)	F4 ± SD (%)
0.042	5.508 ± 0.242	5.684 ± 0.434	5.239 ± 0.316	5.244 ± 0.104
0.167	7.600 ± 0.336	8.431 ± 0.810	8.655 ± 0.921	8.819 ± 0.421
0.333	8.815 ± 1.322	10.838 ± 2.410	11.184 ± 0.932	11.257 ± 1.190
1	15.410 ± 2.937	18.308 ± 5.133	16.890 ± 2.141	17.584 ± 2.639
2	20.975 ± 1.290	22.276 ± 4.221	21.879 ± 3.041	21.733 ± 2.289
3	26.644 ± 2.432	25.869 ± 4.955	25.293 ± 2.381	24.846 ± 3.826
5	35.394 ± 3.062	35.002 ± 7.188	33.671 ± 2.390	34.440 ± 4.312
7	47.619 ± 3.936	47.128 ±10.095	53.481 ± 8.172	48.266 ± 5.266
10	67.127 ± 3.108	68.477 ± 9.020	76.872 ± 0.709	63.102 ±11.623
14	94.138 ± 1.354	94.248 ± 5.743	91.907 ± 1.110	82.524 ± 3.169
18	93.636 ± 1.459	96.515 ± 3.625	89.082 ± 2.035	90.143 ± 6.401

Table S4. Mass changes of MOX-MS in the *in vitro* degradation process (n = 3).

Time (w)	F1 ± SD (%)	F2 ± SD (%)	F3 ± SD (%)	F4 ± SD (%)
0.0417	99.60 ± 2.40	99.00 ± 1.55	98.66 ± 2.81	98.32 ± 6.74
1	98.07 ± 0.93	96.84 ± 1.78	98.36 ± 2.52	99.87 ± 7.90
2	96.13 ± 3.59	94.62 ± 2.72	95.30 ± 0.10	96.82 ± 2.59
4	96.54 ± 1.98	94.20 ± 2.70	87.99 ± 2.50	95.38 ± 0.07
6	96.34 ± 4.08	92.99 ± 2.60	87.56 ± 2.36	90.87 ± 0.19

8	97.65 ± 4.07	92.84 ± 5.38	82.93 ± 2.49	84.54 ± 3.30
12	93.30 ± 5.28	90.52 ± 4.27	80.52 ± 6.65	78.74 ± 7.36
16	88.46 ± 4.45	84.87 ± 2.81	75.72 ± 2.06	49.44 ± 3.31
20	86.52 ± 3.37	82.47 ± 1.28	49.80 ± 1.86	42.24 ± 4.78
24	78.36 ± 2.20	61.33 ± 1.69	34.05 ± 5.63	17.67 ± 7.87

Table S4. Polymer Mw changes of MOX-MS in the *in vitro* degradation process (n = 3).

Time (w)	F1 ± SD (%)	F2 ± SD (%)	F3 ± SD (%)	F4 ± SD (%)
0	100 ± 0	100 ± 0	100 ± 0	100 ± 0
1	101.65 ± 0.12	97.25 ± 0.32	98.03 ± 1.55	97.67 ± 0.11
4	95.56 ± 0.27	94.24 ± 0.26	88.62 ± 0.56	84.39 ± 2.37
8	89.16 ± 1.31	82.50 ± 0.41	63.56 ± 1.63	40.24 ± 2.56
12	81.10 ± 0.11	49.96 ± 0.20	32.51 ± 1.00	12.89 ± 2.31
16	58.93 ± 0.55	37.12 ± 2.17	11.27 ± 0.38	1.87 ± 1.04
20	50.01 ± 2.56	25.74 ± 3.66	3.84 ± 0.70	/
24	10.78 ± 3.63	11.19 ± 1.93	/	/

Table S4. pH changes of release medium in the *in vitro* degradation process (n = 3).

Time (w)	F1 ± SD (%)	F2 ± SD (%)	F3 ± SD (%)	F4 ± SD (%)
0	7.60 ± 0.02	7.60 ± 0.02	7.60 ± 0.02	7.80 ± 0.02
1	7.59 ± 0.01	7.67 ± 0.02	7.57 ± 0.02	7.80 ± 0.01
2	7.58 ± 0.02	7.62 ± 0.05	7.55 ± 0.03	7.61 ± 0.06
4	7.46 ± 0.01	7.53 ± 0.01	7.31 ± 0.03	6.94 ± 0.06
6	7.33 ± 0.01	7.41 ± 0.02	7.15 ± 0.02	6.58 ± 0.01
8	7.23 ± 0.02	7.30 ± 0.05	6.82 ± 0.03	5.92 ± 0.06
12	7.17 ± 0.01	7.20 ± 0.01	6.54 ± 0.03	5.33 ± 0.06
16	7.17 ± 0.01	7.10 ± 0.02	5.34 ± 0.02	4.72 ± 0.01
20	7.14 ± 0.02	6.38 ± 0.05	5.04 ± 0.03	4.57 ± 0.06
24	6.93 ± 0.01	5.50 ± 0.01	4.63 ± 0.03	4.45 ± 0.06

Table S5. Plasma concentration values of moxidectin after a single subcutaneous injection of MOX-MS or moxidectin solution (1 mg/kg) (n = 6).

Time (d)	F1 ± SD (ng/mL)	F2 ± SD (ng/mL)	F3 ± SD (ng/mL)	F4 ± SD (ng/mL)	Solution ± SD (ng/mL)
0.04	24.163 ± 4.482	24.474 ± 2.981	19.715 ± 2.647	16.414 ± 3.227	595.653 ± 92.640
0.08	33.025 ± 6.509	39.313 ± 6.000	20.021 ± 2.810	16.986 ± 3.544	368.847 ± 158.290
0.17	37.923 ± 3.022	44.982 ± 6.59	21.377 ± 2.349	26.052 ± 2.506	238.571 ± 72.282
0.25	44.488 ± 2.984	21.371 ± 2.821	22.773 ± 4.562	16.549 ± 3.217	147.684 ± 46.478
0.33	23.786 ± 9.16	25.640 ± 4.747	25.927 ± 7.387	16.556 ± 1.294	117.951 ± 50.889
0.5	10.77 ± 3.358	14.780 ± 2.128	25.475 ± 3.318	18.357 ± 4.742	85.620 ± 41.119
1	12.137 ± 0.92	11.070 ± 1.462	21.992 ± 2.352	13.247 ± 4.372	55.379 ± 26.923
2	9.951 ± 2.302	9.771± 3.068	20.382 ± 3.578	9.327 ± 2.059	21.365 ± 13.002
3	5.03 ± 0.949	6.67 ± 1.215	6.964 ± 1.915	6.033 ± 2.503	18.747 ± 19.052
5	10.624 ± 4.277	5.246 ± 0.592	12.701 ±1.509	12.141 ± 3.672	14.983 ± 9.767
7	2.462 ± 0.951	2.258 ± 0.349	8.627 ± 2.896	11.764 ±11.806	13.247 ± 10.952
9	3.386 ± 1.669	10.135 ± 4.420	7.015 ± 2.556	5.198 ± 2.994	10.122 ±7.084
12	6.56 ± 0.634	13.214 ± 6.48	7.193 ± 1.512	6.558 ± 0.695	7.127 ± 6.826
15	9.641 ± 3.992	15.828 ± 0.908	11.006 ± 3.705	8.352 ± 6.37	5.106 ± 4.874
20	7.539 ± 0.504	9.349 ± 1.185	14.103 ± 3.792	4.237 ± 2.741	5.489 ± 3.795
25	2.331 ± 0.388	11.09 ± 1.134	11.877 ± 3.361	4.561 ± 1.065	3.102 ± 1.989
30	0.848 ± 0.577	12.127 ± 4.605	12.447 ± 4.746	5.479 ± 2.731	0.899 ± 1.040

35	8.173 ± 1.064	7.366 ± 0.395	9.882 ± 1.295	11.16 ± 5.282	1.731 ± 0.729
40	6.565 ± 3.469	6.030 ± 2.978	10.12 ±1.714	7.193 ± 1.663	2.341 ± 2.484
45	3.451 ± 1.308	5.761 ± 1.007	10.800 ± 1.387	5.700 ± 4.664	1.059 ± 0.506
50	6.121 ± 0.920	3.255 ± 2.538	7.667 ± 1.600	4.543 ± 0.591	0.347 ± 0.497
55	3.132 ± 0.735	4.836 ± 1.738	6.233 ± 3.745	6.125 ± 2.245	1.470 ± 1.132
60	2.000 ± 1.409	2.909 ± 1.351	3.256 ± 1.745	2.908 ± 1.979	0.739 ± 0.593
